# Plasma Alzheimer’s biomarkers and brain amyloid in Hispanic and non-Hispanic older adults

**DOI:** 10.1002/alz.13456

**Published:** 2023-09-06

**Authors:** Breton M. Asken, Wei-En Wang, Karen McFarland, Franchesca Arias, Jacob Fiala, Idaly Velez-Uribe, Robin P. Mayrand, Luana Okino Sawada, Christian Freytes, Michael Adeyosoye, Michael Marsiske, Monica Rosselli, Warren W. Barker, Rosie Curiel Cid, David A. Loewenstein, Steven T. DeKosky, Melissa J. Armstrong, Glenn E. Smith, Malek Adjouadi, David E. Vaillancourt, Ranjan Duara

**Affiliations:** 1 1Florida Alzheimer’s Disease Research Center, Gainesville, Florida, USA; 2 Department of Clinical and Health Psychology, University of Florida, Gainesville, Florida, USA; 3 Department of Applied Physiology and Kinesiology, University of Florida, Gainesville, Florida, USA; 4 Department of Neurology, University of Florida, Gainesville, Florida, USA; 5 Department of Psychology, Florida Atlantic University, Boca Raton, Florida, USA; 6 Department of Electrical and Computer Engineering, Center for Advanced Technology and Education, Florida International University, Miami, Florida, USA; 7 Wien Center for Alzheimer’s Disease and Memory Disorders, Mt. Sinai Medical Center, Miami, Florida, USA; 8 Departments of Psychiatry and Behavioral Sciences and Neurology, Center for Cognitive Neuroscience and Aging, University of Miami, Miami, Florida, USA

**Keywords:** Alzheimer’s, amyloid PET, dementia, ethnicity, GFAP, Hispanic, NfL, plasma biomarkers, p-tau181

## Abstract

**INTRODUCTION:**

Alzheimer’s disease studies often lack ethnic diversity.

**METHODS:**

We evaluated associations between plasma biomarkers commonly studied in Alzheimer’s (p-tau181, GFAP, and NfL), clinical diagnosis (clinically normal, amnestic MCI, amnestic dementia, or non-amnestic MCI/dementia), and A*β*-PET in Hispanic and non-Hispanic older adults. Hispanics were predominantly of Cuban or South American ancestry.

**RESULTS:**

Three-hundred seventy nine participants underwent blood draw (71.9 ± 7.8 years old, 60.2% female, 57% Hispanic of which 88% were Cuban or South American) and 240 completed A*β*-PET. P-tau181 was higher in amnestic MCI (*p* = 0.004, *d* = 0.53) and dementia (*p* < 0.001, *d* = 0.97) than in clinically normal participants and discriminated A*β* PET[+] and A*β*-PET[−] (AUC = 0.86). P-tau181 outperformed GFAP and NfL. There were no significant interactions with ethnicity. Among amnestic MCI, Hispanics had lower odds of elevated p-tau181 than non-Hispanic (OR = 0.41, *p* = 0.006).

**DISCUSSION:**

Plasma p-tau181 informs etiological diagnosis of cognitively impaired Hispanic and non-Hispanic older adults. Hispanic ethnicity may relate to greater likelihood of non-Alzheimer’s contributions to memory loss.

## 1 | BACKGROUND

Dementia diagnoses are more common among Hispanic than non-Hispanic older adults.^1,2^ Alzheimer’s disease (AD) is the most common neuropathological finding in patients with dementia.^3^ Identifying AD during life has become increasingly precise with fluid and neuroimaging biomarkers including cerebrospinal fluid (CSF) and positron emission tomography (PET) measurement of beta-amyloid (A*β*) and phosphorylated tau (p-tau). Several diagnostic AD biomarker tests are now FDA approved, but widespread access remains limited by cost, time, and invasiveness of test procedures. The rapid development of both sensitive and specific plasma-based AD biomarkers has made it likely that many of these issues can be circumvented, thus enabling their use for diagnosis, prognosis, and tracking treatment response.

Plasma tau phosphorylated at residues threonine 181 (p-tau181) and threonine 217 (p-tau217) are the most studied blood-based AD biomarkers. Both have high specificity to AD pathology, good prognostic value across the AD spectrum,^4–6^ and correlate well with A*β* and tau PET as well as with cognitive function.^7–9^ Plasma glial fibrillary acidic protein (GFAP), a marker associated with astrocytic reactivity, is another promising blood-based AD biomarker that relates strongly to A*β* pathology and begins to increase early in the course of AD.^10–12^ Plasma biomarkers also show promise for application in clinical trials, serving to facilitate cost-effective screening prior to enrollment and as biological endpoints.^13–15^ However, the lack of ethnic diversity is a common criticism of prior plasma biomarker studies, hindering understanding of generalizability and broad applicability.

Although existing data are sparse, at least one study has demonstrated elevated plasma p-tau181 and p-tau217 levels in participants who were A*β*-PET positive, as well as in those with neuropathologically confirmed AD in a multiethnic older cohort.^16^ There were no clear differences in biomarker performance across race or ethnicity subgroups, though their sample sizes were relatively small especially for studying plasma biomarkers in comparison to amyloid PET. Additionally, symptoms presumed to be related to underlying AD pathology may differ by ethnicity. One study showed lower rates of positive A*β*-PET scans among Hispanic older adults suspected of having AD clinically.^17^ Clinical translation of plasma AD biomarkers requires continued evaluation in study cohorts that reflect the growing sociocultural and ethnic diversity of the population.

In the present study, we evaluated how plasma p-tau181, GFAP, and a marker of neurodegeneration (neurofilament light chain; NfL) differed by clinical diagnosis and related to A*β*-PET in Hispanic and non-Hispanic older adults from the 1Florida Alzheimer’s Disease Research Center (1FLADRC). Building off emerging data based on A*β*-PET, we then derived a p-tau181 cutoff for “amyloid positivity” based on participants with a corresponding A*β*-PET to evaluate whether the likelihood of a “positive” p-tau181 test differed between Hispanic and non-Hispanic older adults with a diagnosis of amnestic mild cognitive impairment (MCI).

## 2 | METHODS

### 2.1 | Participants

All study participants were enrolled in the 1FLADRC, which includes older adults spanning the continuum of normal cognition, MCI, and dementia. The 1FLADRC cohort is unique due to its ethnic diversity. Over 50% of participants self-identify as Hispanic (mostly Hispanic/Latino, which we will refer to as “Hispanic” collectively throughout), primarily of Cuban (32%) or South American (18%) origin. Participants are recruited from outpatient memory disorders clinics, free memory screening programs, and community outreach. Currently, only a small number of participants self-identifying as Black/African American have completed both blood draw and PET scan (*N* = 6). Therefore, this study focused on Hispanic (HW) and non-Hispanic (NHW) older adults self-reporting as “White” with regards to race.

### 2.2 | Clinical evaluation and medical history

The 1FLADRC participants completed comprehensive neurological and neuropsychological evaluations including elements from the National Alzheimer’s Coordinating Center (NACC) Uniform Data Set (UDS) plus cognitive measures specific to the center. Overall functioning was assessed with the Clinical Dementia Rating (CDR) scale. Hispanic participants were tested in either Spanish or English by bilingual psychometricians. Data for each participant were reviewed during a multidisciplinary consensus conference to specify a clinical diagnosis. For this study, all participants were classified using NACC/UDS-based designations of clinically normal (CN), amnestic-predominant mild cognitive impairment (AMN-MCI), amnestic-predominant dementia (AMN-DEM), non-amnestic MCI or dementia (NONAMN), or cognitively impaired but not MCI (IMPnonMCI). Clinical diagnoses are rendered prior to reviewing biomarker data such as magnetic resonance imaging or PET.

Given prior reports of different rates of medical history risk factors like heart disease(s) between Hispanic and non-Hispanic populations, we describe frequency of multiple cardiovascular medical history factors and reported concurrent medication use from information collected through the UDS Health History. We further calculated a modified vascular burden score (VBS) as a composite metric of brain vascular risk as described previously^18^ but with two additional components. Briefly, the VBS in our study represents a sum of seven self-reported risk factors or diagnoses: cardiac-arrhythmias (atrial fibrillation OR defibrillator), coronary artery disease (angina OR angioplasty/endarterectomy/stent OR cardiac bypass OR heart attack), congestive heart failure, cerebrovascular disease (stroke OR transient ischemic attack), hypertension, hypercholesterolemia, diabetes (Type I or Type II). Higher VBS represents greater risk for vascular contributions to cognitive impairment.

### 2.3 | Plasma biomarkers

Duplicate blood samples were analyzed in-house (Quanterix SRX analyzer), blinded to all clinical and demographic data, using single molecule array (Simoa) technology for p-tau181 (Quanterix Advantage v2 kit), GFAP, and NfL (Quanterix Neurology 2-Plex B). Our group previously reported separate plasma NfL data in a smaller sample of the 1FLADRC.^19^ NfL data are reported here in a larger cohort with different study aims and using a different Quanterix Simoa assay. Samples with coefficients of variation >20% were excluded (p-tau181: 10.8% of sample, GFAP: 7.4%, NfL: 6.3%). Additional sample collection and processing details are provided in Supplementary Methods.

### 2.4 | Amyloid-PET imaging

A*β*-PET was performed with either [18F] florbetaben (FBB; 90% of scans) or [18F] florbetapir (FBP; 10% of scans). PET imaging protocols are described further in Supplementary Methods. For quantification, we calculated a global composite standardized uptake value ratio (SUVR; cerebellar grey matter reference) and converted to a Centiloid (CL) scale.^20^ A*β*-PET scans were classified as either positive (A*β*-PET[+]) or negative (A*β*-PET[−]) by a trained reader following manufacturer interpretation protocols. A small number of participants (3%) in the dataset were initially without a visual read determination and were classified as A*β*-PET[+] or A*β*-PET[−] based on quantitative CL thresholds established for the 1FADRC (FBB SUVR ≥ 1.42, FBP SUVR ≥ 1.20, CL ≥ 29). Visual reads were later obtained and all were consistent with quantification-based classification. All A*β*-PET scans were obtained within 1 year of blood draw except for six participants who underwent A*β*-PET imaging >1 year after blood draw (range 405 to 798 days) and were A*β*-PET[−] at that time.

### 2.5 | *APOE* genotyping

All samples for *APOE* genotyping were performed in Dr. Nilüfer Ertekin-Taner’s laboratory (Mayo Clinic, Jacksonville, Florida, USA); the *APOE ɛ*2, *ɛ*3, and *ɛ*4 alleles used predesigned TaqMan SNP Genotyping Assays for SNPs rs7412 and rs429358 (Thermo Fisher Scientific, Massachusetts, USA) on the QuantStudio 7 Flex Real-Time PCR system (Applied Biosystems, California, USA) following the manufacturer’s protocol.

### 2.6 | Statistical analyses

Data were analyzed using SPSS v28. Plasma biomarker data were log-transformed. We used analysis of covariance (ANCOVA) adjusting for age with Tukey-Kramer post hoc analyses to compare biomarker concentrations among five clinical diagnostic groups (CN, AMN-MCI, AMN-DEM, NONAMN, IMPnonMCI). To analyze the association between plasma markers and A*β*-PET, we evaluated (a) how biomarker concentrations related to A*β* burden measured in CLs (Spearman’s rho), (b) group differences between A*β*-PET[+] and A*β*-PET[−] (ANOVA), and (c) discriminability (area under the receiver operative characteristics curve [AUC/ROC]). Combined performance of plasma biomarkers for discriminating A*β*-PET[+] and A*β*-PET[−] was evaluated using binary logistic regression and odds ratios (ORs) with and without adjustment for a base model including participant age, sex, and *APOE ɛ*4 carrier status. We investigated factors potentially influencing the accuracy of A*β*-PET prediction by comparing correctly and incorrectly classified participants on age, sex, ethnicity, and *APOE ɛ*4 carrier status (ANOVA and chi-square). Interactions between independent variables of interest and ethnicity were evaluated for all analyses to inform whether the observed relationships were significantly different between HW and NHW participants.

Lastly, we aimed to build on prior work that used A*β*-PET^17^ to determine whether plasma p-tau181 differed between HW and NHW older adults diagnosed with amnestic-predominant cognitive decline, the most common clinical phenotype of AD. We focused on individuals diagnosed with AMN-MCI (the largest study group). A Youden’s Index cutoff that optimized balance of sensitivity (Sens) and specificity (Spec) to A*β*-PET[+] participants was derived from the cohort that underwent both blood draw and A*β*-PET. Using this cutoff, we created putative AD[+] and AD[−] groups based on plasma p-tau181 (plasmaAD[+] and plasmaAD[−]) among study participants who contributed blood regardless of whether they had a PET scan. Among those diagnosed with AMN-MCI, we used logistic regression to analyze differences in the likelihood of being plasmaAD[+] between ethnic groups.

For all analyses, we used an a priori alpha of *p* < 0.05 (two-sided) and report effect size estimates and/or 95% confidence intervals. We interpreted effect size estimates as follows: Cohen’s *d* (0.2 to 0.5 = small, 0.5 to 0.8 = medium, > 0.8 = large), eta squared (*η*^2^; 0.01 to 0.04 = small, 0.06 to 0.11 = medium, >0.11 = large), AUC (0.60 to 0.69 = poor, 0.70 to 0.79 = fair, 0.80 to 0.89 = good, 0.90 to 1.00 = excellent), Pearson’s *r* and Spearman’s rho (0.1 to 0.3 = small, 0.3 to 0.5 = medium, >0.5 = large).

## 3 | RESULTS

### 3.1 | Sample characteristics

The plasma biomarker cohort included 379 older adults (age 71.9 ± 7.8 years, 60.2% female, 56.9% HW, 15.2 ± 3.5 years of education; Table 1). The HW and NHW subgroups did not significantly differ in age, frequency of *APOE ɛ*4 carriers, or CDR Sum of Boxes. The HW participants reported fewer years of education (14.4 ± 3.7 vs 16.3 ± 3.0, *p* < 0.001) and had a greater proportion of females (64.8% vs 54.0%, *p* = 0.03). Among CN participants, higher plasma concentrations of GFAP (*ρ* = 0.52 [0.29 to 0.69], *p* < 0.001) and NfL (*ρ* = 0.44 [0.20 to 0.63], *p* < 0.001), but not p-tau181 (*ρ* = 0.19 [−0.09 to 0.43], *p* = 0.17) were significantly associated with older age. The three markers correlated strongly with each other (all *ρ*= 0.52 to 0.58, all *p* < 0.001; see Supplementary Results eFigure).

Overall VBS did not significantly differ between groups (*p* = 0.17). Some cardiovascular factors were at least modestly more frequent (chi-square, *p* < 0.20) in NHW (angioplasty/endarterectomy/stent, pacemaker/defibrillator, angina), and some were at least modestly more frequent in HW (heart attack, diabetes, hypertension, hypercholesterolemia). Regarding medications, HW were more likely to report concurrent use of angiotensin II inhibitors (26.5% vs 13.6%, *p* = 0.002); Supplementary Results eTable 1).

### 3.2 | Plasma markers by clinical diagnosis

**FIGURE 1:**
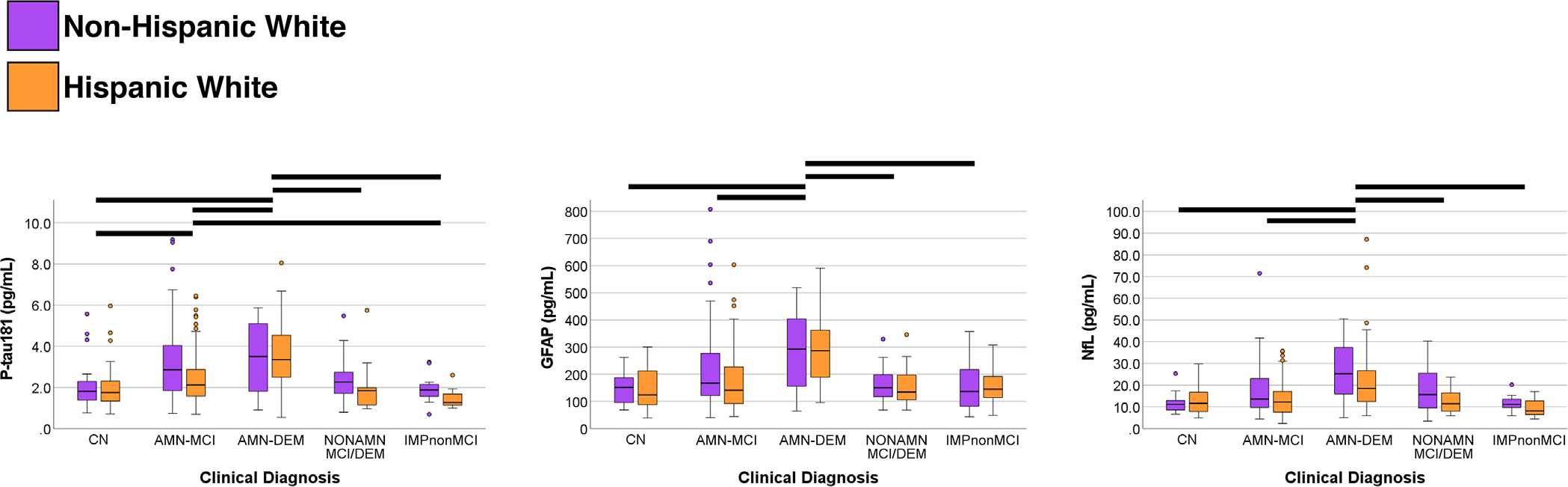
Plasma biomarker concentration differences between clinical diagnostic groups for p-tau181, GFAP, and NfL. For p-tau181, participants diagnosed with AMN-DEM had significantly higher concentrations than all other groups (black lines), and those with AMN-MCI had higher concentrations than CN and IMPnonMCI. For both GFAP and NfL, only participants with AMN-DEM had significantly higher concentrations than other diagnostic groups. Findings did not differ between ethnicity groups. Four participants with very high plasma NfL (> 100 pg/mL, *N* = 3 AMN-DEM, *N* = 1 NONAMN) are not shown visually in the figure to avoid extensive y-axis distortion. AMN-DEM, amnestic dementia; AMN-MCI, amnestic MCI; CN, normal controls; GFAP, glial fibrillary acidic protein; IMPnonMCI, cognitively impaired but not MCI; MCI, mild cognitive impairment; NfL, neurofilament light; NONAMN, non-amnestic MCI or dementia

We compared plasma biomarker concentrations between clinical diagnosis groups: CN (*N* = 62), AMN-MCI (*N* = 179), AMN-DEM (*N* = 58), NONAMN (*N* = 48), IMPnonMCI (*N* = 28); see Supplementary Results eTable2. There were statistically significant differences between clinical diagnosis groups for plasma p-tau181 (*p* < 0.001, *η*^2^ = 0.125), GFAP (*p* < 0.001, *η*^2^ = 0.130), and NfL (*p* < 0.001, *η*^2^ = 0.127). Pairwise post hoc comparisons showed that AMN-DEM had significantly higher p-tau181 concentrations than all other diagnostic groups and AMN-MCI had higher p-tau181 than CN and IMPnonMCI (Figure 1). For both GFAP and NfL, only AMN-DEM significantly differed (higher) from the other diagnostic groups. There were no statistically significant interactions between clinical diagnosis and ethnicity for any plasma biomarker.

### 3.3 | Plasma biomarkers and A*β*-PET

**FIGURE 2:**
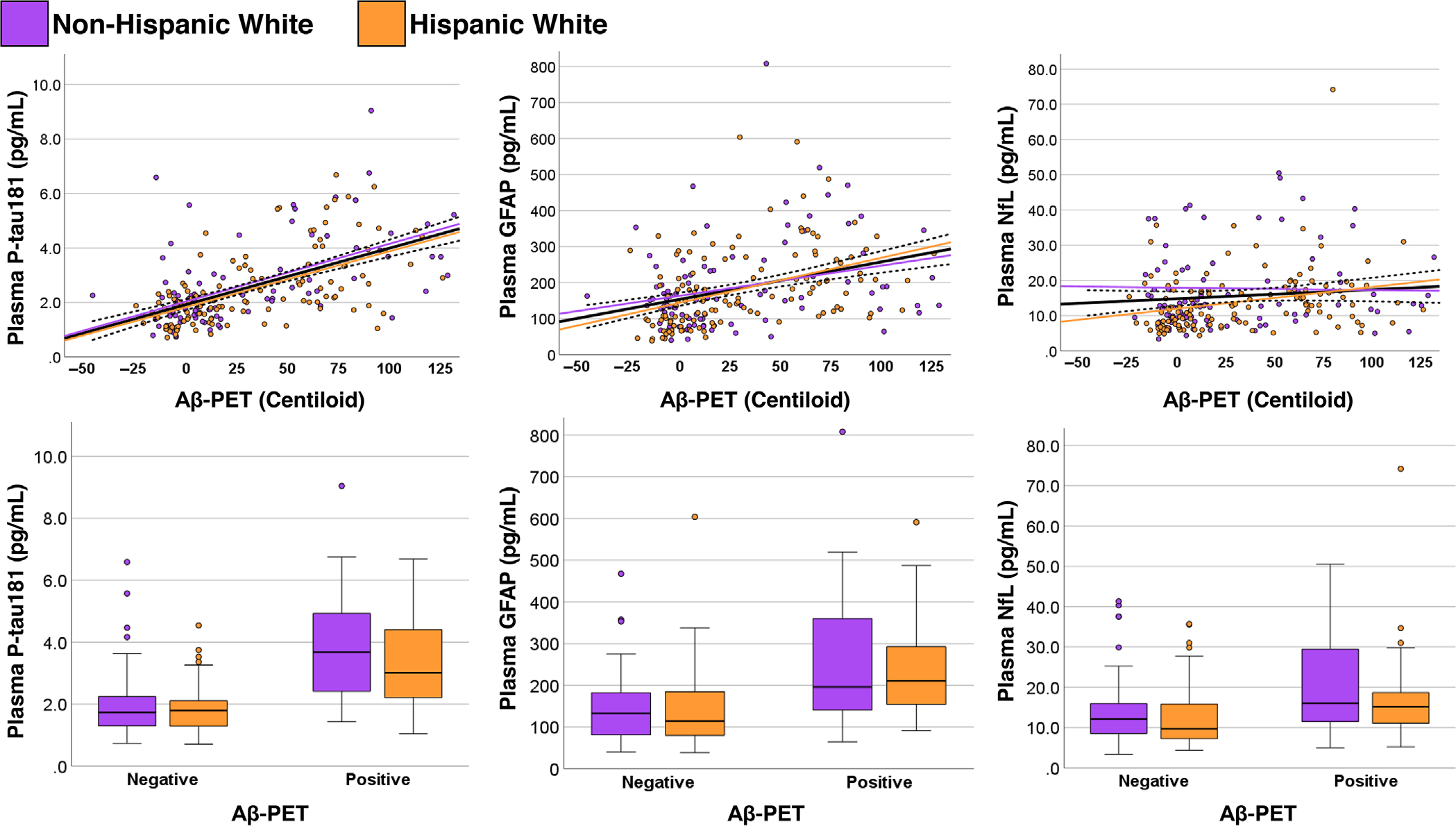
Association between plasma biomarker concentrations and cortical A*β* burden. Data are shown in relation to a continuous measure of A*β* burden quantified on the Centiloid scale (top row), and between participants classified as having a positive or negative scan (bottom row). For the associations with the Centiloid scale, statistically significant associations were observed in the overall sample (black line) for all biomarkers, but effect sizes were notably larger for plasma p-tau181 (*ρ* = 0.59 [0.50 to 0.67]) and GFAP (*ρ* = 0.42 [0.30 to 0.52]) than for NfL (*ρ* = 0.23 [0.10 to 0.35]). Results were similar when comparing participants based on dichotomous (positive or negative) A*β*-PET status. The strength of association between plasma markers and A*β*-PET outcomes did not significantly differ between Hispanic (orange) and Non-Hispanic (purple) ethnicity groups. A*β*, beta-amyloid; GFAP, glial fibrillary acidic protein; NfL, neurofilament light; PET, positron emission tomography

A total of 240 participants underwent A*β*-PET (63% of the overall sample). The cohort with A*β*-PET did not differ significantly in demographics or disease severity (CDR) from the overall sample. Higher concentrations of all three plasma biomarkers were significantly associated with greater A*β* burden (Figure 2). The strongest relationship was seen for plasma p-tau181 (*ρ*= 0.59 [0.50 to 0.67], *p* < 0.001), followed by GFAP (*ρ*= 0.42 [0.30 to 0.52], *p* < 0.001) and NfL (*ρ*= 0.23 [0.10 to 0.35], *p* < 0.001). A similar pattern was observed when making the dichotomous comparison between A*β*-PET[+] and A*β*-PET[−] participants. Namely, plasma p-tau181 had the strongest group difference (*p* < 0.001, *d* = 1.3), followed by GFAP (*p* < 0.001, *d* = 1.00) and NfL (*p* < 0.001, *d* = 0.55). There were no statistically significant interactions between A*β*-PET status and ethnicity for any plasma biomarker.

**FIGURE 3:**
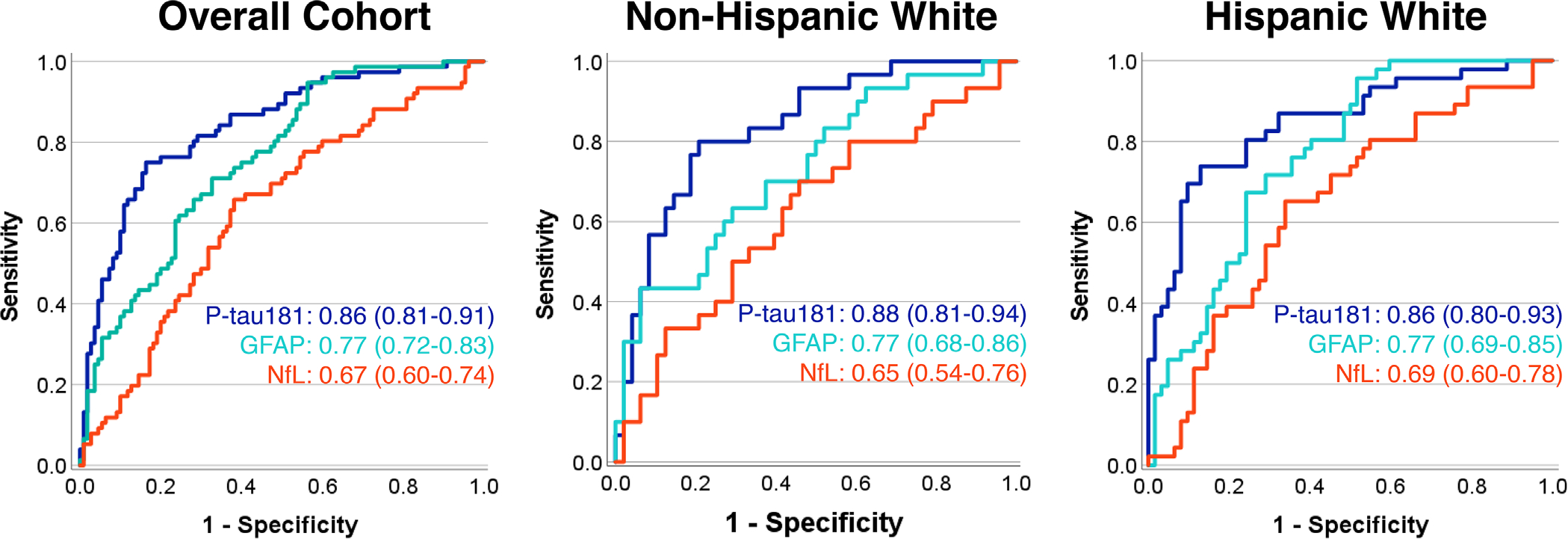
Area under the curve analysis demonstrating discriminability between A*β*-PET positive and negative participants for each plasma biomarker. Data are shown for the overall cohort, Non-Hispanic participants, and Hispanic participants. Plasma p-tau181 concentrations most accurately discriminated A*β*-PET positive and negative participants (maroon) followed by GFAP (blue) and NfL (green). Discrimination accuracy was similar for both ethnicity groups. A*β*, beta-amyloid; GFAP, glial fibrillary acidic protein; NfL, neurofilament light; PET, positron emission tomography

Plasma p-tau181 concentrations had good discriminability between A*β*-PET[+] and A*β*-PET[−] participants (AUC = 0.85 [0.79 to 0.90], Youden’s Index = 2.39 pg/mL, Sens = 75%, Spec = 85%; Figure 3). Plasma GFAP had fair discriminability (AUC = 0.77 [0.71 to 0.83], Youden’s Index = 170 pg/mL, Sens = 69%, Spec = 71%), and plasma NfL had poor discriminability (AUC = 0.67 [0.60 to 0.74], Youden’s Index = 12.86 pg/mL, Sens = 67%, Spec = 66%). The combined discriminability of the two top performing markers (p-tau181 and GFAP) was then evaluated with logistic regression. Plasma p-tau181 significantly improved discriminability between A*β*-PET[+] and A*β*-PET[−] participants beyond the base model of age, sex, and *APOE ɛ*4 carrier (ΔR^2^ = 0.26, OR = 3.0 [2.0 to 4.5], *p* < 0.001). Adding plasma GFAP did not significantly improve discriminability beyond p-tau181 (ΔR^2^ = 0.02, *p* = 0.07). Plasma biomarker discriminability of A*β*-PET[+] and A*β*PET[−] participants did not significantly differ between HW and NHW participants.

### 3.4 | Misclassification analysis

Plasma p-tau181 alone misclassified A*β*-PET status for 45 (22%) participants with a tendency towards false negatives (ie, 33/45 were observed A*β*-PET[+] but predicted to be A*β*-PET[−] based on P-tau181). Misclassified participants did not significantly differ from correctly classified participants in age, sex, or ethnicity. Among those with available *APOE* genotyping (~90% of sample), an *APOE ɛ*4 allele was more frequently present among misclassified (54%) than correctly classified participants (36%; *p* = 0.04). We further evaluated whether A*β* burden was associated with classification accuracy. Among A*β*-PET[−] participants, there were no significant differences in CLs between true negative and false positive participants. Among A*β*PET[+], there was also no significant difference in CLs between true positive and false negative participants.

### 3.5 | Plasma AD biomarkers in amnestic (AMN)-MCI

There were 161 participants diagnosed with AMN-MCI (*N* = 65 NHW, *N* = 96 HW). Applying the Youden’s Index cutoff of 2.39 pg/mL derived from the A*β*-PET cohort, 73 (45.3%) were classified as plasmaAD[+] (ie, plasma p-tau181 > 2.39pg/mL) and 88 (54.7%) as plasma AD[−]. HW older adults with AMN-MCI had significantly lower odds of being plasmaAD[+] than NHW older adults with AMN-MCI (36.5% vs 58.5%; OR = 0.41 [0.21 to 0.78], *p* = 0.006). Results remained significant after controlling for age and sex (OR = 0.45 [0.23 to 0.87], *p* = 0.02), and were slightly attenuated when further including *APOE ɛ*4 carrier status in the model (OR = 0.52 [0.25 to 1.1], *p* = 0.08). When restricting the analysis to the subset of 61 participants with plasma only who did not contribute to the plasmaAD cutoff derivation (*N* = 25 NHW, *N* = 36 HW), there remained lower odds of HW being plasmaAD[+], but the effect was not statistically significant due to the smaller sample (OR = 0.39 [0.14 to 1.1], *p* = 0.08), and again slightly attenuated when further adjusting for age and sex (OR = 0.49 [0.16 to 1.5], *p* = 0.2) and *APOE ɛ*4 (OR = 0.53 [0.15 to 1.8], *p* = 0.3). To explore whether vascular disease risk contributed to discrepancies between plasmaAD status and AMN-MCI diagnoses (ie, cerebrovascular drivers of memory loss), we compared overall VBS scores between plasmaAD[+] and plasmaAD[−] participants with AMN-MCI (Mann-Whitney *U*). No significant differences were observed for the overall cohort or within the HW or NHW groups separately (all *p* > 0.5).

## 4 | DISCUSSION

We evaluated how plasma biomarkers of AD (p-tau181), astrocyte reactivity (GFAP), and neurodegeneration (NfL) related to clinical diagnosis and A*β*-PET in a large cohort of HW and NHW older adults. Plasma p-tau181 was significantly elevated in both AMN-MCI and AMN-DEM and showed the strongest association with A*β*-PET. GFAP and NfL differed only in the AMN-DEM group, were modestly associated with A*β*-PET, and did not improve the discriminability of A*β*-PET[+] and A*β*-PET[−] participants beyond plasma p-tau181. We found that misclassifying A*β*-PET status based on plasma biomarkers was more likely in *APOE ɛ*4 carriers (commonly false negatives) but not associated with age, sex, ethnicity, or A*β* burden. Importantly, ethnicity did not significantly influence biomarker performance, consistent with our group’s prior work focused on plasma NfL.^19^ Our findings are among the strongest evidence to date that plasma AD biomarkers have diagnostic utility and adequately reflect AD pathology among both HW and NHW older adults. However, among participants with AMN-MCI, we saw that HW participants had a lower odds of positive AD biomarkers based on a p-tau181, suggesting higher rates of non-AD etiology(ies) underlying memory loss.

Plasma biomarkers were highest in amnestic-predominant clinical phenotypes, but there was significant variability within groups. This likely reflects an enrichment for, but not universal presence of, AD as a primary etiology among the amnestic phenotypes.^21,22^ Neuropathological heterogeneity among patients with memory loss underscores the importance of AD biomarkers, such as plasma p-tau181, for improving accuracy of the etiological differential. Our understanding of clinical syndromes across neurodegenerative diseases like AD inadequately accounts for the possibility that symptoms may manifest differently across ethnocultural groups. This may partially explain recent findings that Hispanic older adults diagnosed with MCI or dementia due to *suspected* AD were less likely than NHW to have a positive A*β*-PET scan.^17^ Our findings are similar when using plasma p-tau181. We contend that integrating AD biomarker measurement into ethnically diverse populations is especially important for better characterizing the spectrum of symptoms potentially related (or unrelated) to AD and understanding whether clinicopathological associations differ across underrepresented patient populations. Further, higher rates of medical comorbidities, such as renal and cardiovascular disease, in underrepresented populations may have important implications for the interpretation and utility of plasma biomarkers.^23,24^ We did not observe a higher rate of vascular risk factors or diagnoses in our AMN-MCI participants with negative AD biomarkers, but further research with more direct measures of cerebrovascular disease burden is required.

Our data add to the growing evidence for plasma p-tau181 as a proxy for amyloid-mediated brain pathology across ethnicities,^16^ with the unique feature in this study of having a predominantly Hispanic population. The AUC for differentiating A*β*-PET positive and negative older adults was 0.85, relatively similar to a prior study of p-tau181 (0.77) and p-tau217 (0.84) in a multiethnic cohort with autopsy validation^16^ and other investigations comparing to A*β*-PET but with less ethnically representative cohorts.^6,7,25^ Despite the cost and time burden, A*β*-PET has been used routinely in recent years to screen patients for clinical trials testing of disease-modifying (eg, amyloid-lowering) therapies. The generalizability of results from phase 3 trials for both aducanumab and lecanemab, the first agents of their kind to be FDA-approved for treating patients with AD, along with the recent positive phase 3 trial results for donanemab, suffered from poor racial and ethnic representation.^26–28^ This study importantly demonstrates that continued validation of blood-based tools in multiethnic cohorts may considerably facilitate and reduce the cost of screening representative populations for the purpose of identifying amyloid-positive participants who could fulfill selection criteria for AD clinical trials.

We did not identify obvious factors that predicted misclassification of A*β*-PET status using plasma p-tau181. Most misclassified participants were false negatives, which suggests that the derived threshold for plasma p-tau181 may have been too conservative *in relation to* A*β*-PET positivity in this cohort. *APOE ɛ*4 carriers were slightly overrepresented in the misclassified participants. Genome-wide association studies demonstrate links between *APOE* genotype and plasma p-tau181 regulation,^29,30^ but these were based on NHW participants. Associations between *APOE* status and AD risk are not universal across racial and ethnic groups.^31,32^ Our sample comprised a higher percentage of *APOE ɛ*4 carriers within Hispanic participants of varying ancestries than other studies,^33,34^ which may reflect differences in recruitment sources and a bias towards presence of cognitive impairment in tertiary clinics typical of ADRC populations. Findings may not generalize to patients evaluated in general medicine clinics or at a general population level. Future work should aim to understand whether polygenetic factors linked to variability in ancestral origins influence agreement between AD biomarker modalities (eg, PET vs CSF vs blood) and associations with AD pathology, and whether and how such relationships are consistent across racial and ethnic groups.

Valid blood tests for AD offer the potential of removing barriers to diagnosis associated with more invasive and costly tests, including CSF and PET biomarkers. Persuasive arguments have been made for incorporating plasma AD biomarkers into routine clinical care for diagnostic and prognostic value.^4,35,36^ Efforts to fully automate and standardize plasma assays will facilitate clinical translation and interpretability of proposed cutoff values in different contexts of use.^35^ While our data provide some assurances that stratification or separate cutoffs related to Hispanic ethnicity alone are unnecessary, it is critical to continue prioritizing sociodemographic representation during biomarker development and validation stages. There are likely complex interactions among AD pathology, AD biomarkers, social determinants of brain health, and genetic variability that differ across racial and ethnic groups.^37–39^

Neither plasma GFAP nor NfL improved upon p-tau181 for identifying significant levels of cortical A*β* burden but may still represent important and independent aspects of AD or other neurodegenerative pathophysiology. Plasma GFAP is considered a marker of astrocyte reactivity and appears closely linked to early A*β*-related pathology, but not tau pathology, in AD.^40^ Plasma NfL is a nonspecific marker of neuronal degeneration or injury that only modestly correlates with AD pathology, but could provide complementary information about disease severity, rates of atrophy, or presence of significant co-pathology.^7,19,41^ Ultimately, a blood-based biomarker panel that informs multiple components of neurodegenerative disease(s) will optimize the in vivo characterization of older adults experiencing cognitive and/or behavioral decline as they age.

### 4.1 | Limitations

Our study characterized plasma p-tau181, GFAP, and NfL in relation to clinical diagnosis, A*β*-PET, and atrophy in a unique sample comprised of over 50% Hispanic older adults. However, our cohort had very limited racial diversity, so we focused instead on Hispanic and non-Hispanic older adults self-identifying as White in terms of race. Data were from one multi-institution ADRC and were cross-sectional. Other p-tau epitopes more strongly relate to AD pathology and may have better utility at different points in the disease process (eg, p-tau217, p-tau231).^42–45^ While the plasma biomarker assays used to test our samples are common and commercially available, raw values and cutoffs do not necessarily translate to other studies and are not directly comparable since these are not fully standardized, automated, and Clinical Laboratory Improvements Amendments-approved tests. We did not have neuropathological data for this cohort, which limits conclusions about specificity of our findings to AD pathology. We also do not know details about the expected high likelihood of mixed neuropathology across our sample including those with positive A*β*-PET, nor did we have detailed information about renal function (eg, estimated glomerular filtration rate), factors which may influence interpretation of plasma biomarker values^23,46^ but also represent risk factors for cognitive decline. Angiotensin II inhibitors can be used in treating patients with chronic kidney disease, and reported use was more common in HW than NHW individuals in our sample, but we cannot say with certainty the reasons for self-reported prescribed medications in our sample. The Hispanic older adults in our sample mostly reported family origins in Cuba or South America and results may not generalize to other regions of Hispanic origin that may have different genetic or social determinants of health considerations. Other cohorts such as the Washington Heights-Inwood Columbia Aging Project (WHICAP),^16^ the Health & Aging Brain Among Latino Elders (HABLE),^47^ and Health and Aging Brain Study-Health Disparities (HABS-HD)^41^ will contribute significantly to advancing AD-related research in Hispanic populations.

## 5 | CONCLUSIONS

Plasma biomarkers reflecting AD pathology (p-tau181, GFAP, NfL) may aid etiological diagnosis of cognitively impaired older adults from Hispanic and non-Hispanic ethnic origins. Hispanic ethnicity alone does not significantly influence the interpretation of how plasma p-tau181, GFAP, and NfL relate to A*β*-PET, but may be linked to greater likelihood of non-AD causes of memory loss. Blood-based biomarkers could help reduce barriers to clinical diagnosis and research participation that disproportionately impact underrepresented sociodemographic groups.

## Supplementary Material

Supplementary

## Figures and Tables

**TABLE 1:** Sample characteristics for the overall study sample (*N* = 379) with plasma biomarker data and for the sub-cohort who underwent both blood draw and positron emission tomography imaging (*N* = 240, 63% of overall sample)

	Overall	Hispanic White	Non-Hispanic White	*P*-value
***N***	379	216	163	
Age, years	71.9 (7.8)	71.3 (7.9)	72.7 (7 6)	0.07
Sex, %female	228/379 (60.2)	140/216 (64.8)	88/163 (54.0)	0.03
Education, years	15.2 (3.5)	14.4 (3.7)	16.3 (3.0)	<0.001
CDR global, *N* (%)				0.11
0	93/379 (24.5)	58/216 (26.9)	35/163 (21.5)	
0.5	224/379 (59.1)	116/216 (53.7)	108/163 (66.3)	
1+	62/379 (16.4)	42/216 (18.5)	20/163 (12.2)	
CDR-sum of boxes	2.1 (3.2)	2.3 (3.5)	1.9 (2.6)	0.15
*APOE* ε4, %carrier^a^	116/343 (33.8)	73/206 (35.4)	43/137 (31.4)	0.44
MMSE	26.7 (4.3)	26.3 (4.5)	27.2 (4.0)	0.06
Hispanic/Latino origin,%	-		-	
Cuban	122/379 (32.2)	122/216 (56.5)	-	-
South American	69/379 (18.2)	69/216 (31.9)	-	-
Puerto Rican	10/379 (2.6)	10/216 (4.6)	-	-
Central American	6/379 (1.6)	6/216 (2.8)	-	-
Other	9/379 (2.4)	9/216 (4.2)	-	-
Primary Spanish language	188 (49.6)	188 (87)	0 (0)	-
*Plasma* + *Aβ-PET Cohort*				
*N*	240	135	105	
Age, years	70.9 (7.6)	70.3 (7.2)	71.6 (8.0)	0.16
Sex, %female	143/240 (59.6)	91/216 (67.4)	52/105 (49.5)	0.005
Education, years	15.6 (3.2)	14.9 (3.3)	16.4 (2.9)	<0.001
CDR Global, *N* (%)				0.31
0	58/240 (24.2)	37/135 (27.4)	21/105 (20.0)	
0.5	137/240 (57.1)	71/135 (52.6)	66/105 (62.9)	
1+	45/240 (18.7)	27/135 (20.0)	18/105 (17.1)	
CDR-sum of boxes	2.1 (2.6)	2.2 (2.9)	2.0 (2.2)	0.59
*APOE* ε4, %carrier^a^	86/222 (38.7)	56/133 (42.1)	30/89 (33.7)	0.21
MMSE	26.6 (4.4)	26.3 (4.6)	27.0 (4.0)	0.20
Centiloids	30.0 (39.4)	31.4 (36.9)	28.3 (42.5)	0.55
Hispanicorigin, *N* (%)				
Cuban	74/240 (30.4)	74/135 (54.1)	-	-
South American	46/240 (19.2)	46/135 (34.1)	-	-
Puerto Rican	8/240 (3.3)	8/135 (5.9)	-	-
Central American	5/240 (2.1)	5/135 (3.7)	-	-
Other	3/240 (1.3)	3/135 (2.2)	-	-

*Note*: Data are presented as mean (standard deviation) unless otherwise specified.

Abbreviations: APOE, apolipoprotein E; CDR, Clinical Dementia Rating scale, MMSE, Mini-Mental State Examination.

a 343/379 (91%) of the overall sample and 222/240 (92.6%) of the PET cohort had *APOE* genotyping available.

## Highlights

- Alzheimer’s biomarkers were measured in Hispanic and non-Hispanic older adults.
- Plasma p-tau181 related to amnestic cognitive decline and brain amyloid burden.
- AD biomarker associations did not differ between Hispanic and non-Hispanic ethnicity.
- Hispanic individuals may be more likely to have non-Alzheimer causes of memory loss.

## RESEARCH IN CONTEXT

1. **Systematic review**: Existing research on Alzheimer’s biomarkers primarily includes White, non-Hispanic older adults. We evaluated plasma and positron emission tomography (PET) Alzheimer’s biomarkers in Hispanic and non-Hispanic individuals from the 1Florida Alzheimer’s Disease Research Center.
2. **Interpretation**: Plasma p-tau181, more so than glial fibrillary acidic protein (GFAP) and neurofilament light (NfL), was associated with amnestic cognitive decline and brain amyloid burden. Hispanic older adults with amnestic cognitive decline were less likely than non-Hispanic older adults to have elevated plasma p-tau181, suggesting higher rates of non-Alzheimer’s related impairment.
3. **Future directions**: Plasma p-tau181 may aid etiological diagnosis of cognitively impaired older adults from Hispanic and non-Hispanic ethnic origins. Longitudinal follow-up and identifying non-Alzheimer’s contributions to cognitive decline in ethnically diverse cohorts is critical.
