# Supplementary material for: Plasma Alzheimer’s biomarkers and brain amyloid in Hispanic and non-Hispanic older adults
Source: Alzheimers Dement. Author manuscript; Available in PMC 2024 Feb 14. (PMC10865106; doi:10.1002/alz.13456)
Supplement: Supplementary file 1 [file NIHMS1962752-supplement-Supplementary.pdf]

Supplementary Methods

Plasma Collection and Processing

Venous blood was collected using 10mL Purple Top blood tubes, mixed by inversion 10 times, centrifuged at room temperature for 12 minutes at 1200rcf within 1 hour of collection. 500 microliter aliquots of plasma were then placed in freezer boxes and stored at -80C. Prior to analysis, samples were thawed (1 freeze-thaw cycle) at room temperature, vortexed for 30 seconds, and placed on ice until centrifuging at 10,000g for 5 minutes at 4oC. Samples were run in duplicate using 1:4 dilution following manufacturer recommended protocols for the Quanterix P-tau181 Advantage Kit v2 and the Quanterix 2-plex B kit. All samples were analyzed in-house at the UF Center for Translational Research in Neurodegenerative Diseases (Quanterix SRX analyzer). Samples with coefficients of variation > 20% were excluded from analyses (P-tau181: 10.8% of sample, GFAP: 7.4%, NfL: 6.3%). Mean (SD) of CV%s for included samples was: P-Tau181 (7.1±4.8%), GFAP (7.6±4.9%), NfL (7.1±4.8%).

MRI and Amyloid PET Acquisition and Processing

1Florida ADRC PET scanners (Philip Gemini or Siemens Biograph) and imaging protocols have been approved by ANDI or SCAN. All subjects underwent a 20-min PET scan starting at least 50-90 min after intravenous injection of radiotracers. Florebetaben (F-18 Neuraceq; 90% of scans) or Florebetapir (F-18 Amyvid; 10%) was used. PET scans were reconstructed using Ordered Subsets Expectation Maximization (OSEM) algorithm using 4 iterations and 16 subsets or comparable reconstruction. Acquired PET scan were reconstructed into a 128×128×90 (axial) matrix with voxel dimensions of 2×2×2 mm. Reconstruction was performed using manufacturer-supplied software and included corrections for attenuation, scatter, random coincidences and dead time. Images were smoothed with a 5 mm Gaussian filter. Following reconstruction, image sets were visually inspected.

The amyloid PET scans were coregistered linearly with 12 degrees of freedom, onto subject's T1 weighted MPRAGE scan using FSL toolbox (fsl.fmrib.ox.ac.uk) (Jenkinson, Beckmann, Behrens, Woolrich, & Smith, 2012). This registration process ensured that the PET image had the same accurate segmentation and parcellation as in the MRI. The segmented MRIs from the FreeSurfer pipeline and the co-registered PET images were used to extract the average intensity of individual ROIs, yielding the regional standardized uptake values (SUVs). Regional SUV ratios (SUVRs) were computed by dividing the regional SUVs by the SUV obtained from the cerebellar grey matter. A composite SUVR was calculated by the mean SUVR of the 5 cortical regions (frontal, temporal, parietal, anterior and posterior cingulate cortex regions, each region averaged from left and right hemispheres). SUVRs were converted to a Centiloids (CL) scale where "0" represents mean uptake in healthy young controls devoid of amyloid pathology and "100" represents the typical degree of cortical amyloid deposition observed in PET imaging in patients diagnosed with mild-moderate dementia due to Alzheimer's disease (Klunk et al., 2015). Aβ-PET scans for most participants were classified as either positive (Aβ-PET [+]) or negative (Aβ-PET [-]) by a trained reader following manufacturer interpretation protocols and blinded to clinical and demographic information. A small number of participants (3%) without a visual read determination were

initially classified as Aβ-PET [+] or Aβ-PET [-] based on quantitative CL thresholds established for the 1Florida ADRC (FBB SUVR $\geq$ 1.42, FBP SUVR $\geq$ 1.20, CL $\geq$ 29). These scans were re-evaluated visually, blinded to quantification, and visual reads were consistent with quantification methods.

All Aβ-PET scans were obtained within 1 year of blood draw except for 6 participants who underwent Aβ-PET imaging > 1 year after blood draw (range 405-798 days) and were Aβ-PET [-]. These participants remained in analyses given that we can reasonably conclude the negative scan obtained > 1 year after blood draw would also have been negative at the earlier date of the blood draw.

APOE Genotyping

All samples for *APOE* genotyping were performed in Dr. Nilüfer Ertekin-Taner's laboratory (Mayo Clinic, Jacksonville, FL, USA); the *APOE ε2, ε3*, and *ε4* alleles used predesigned TaqMan SNP Genotyping Assays for SNPs rs7412 and rs429358 (Thermo Fisher Scientific, MA, USA) on the QuantStudio 7 Flex Real-Time PCR system (Applied Biosystems, CA, USA) following the manufacturer's protocol.

Supplementary Results

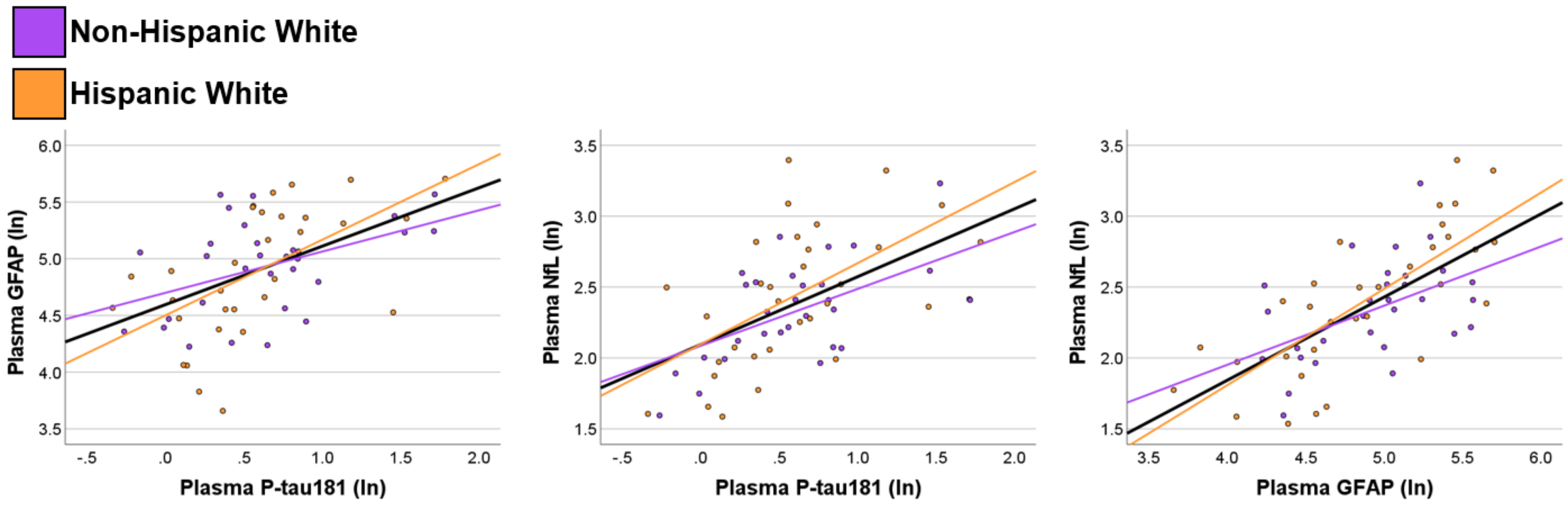

eFigure: Correlations between plasma P-tau181, GFAP, and NfL. Data shown for the overall sample (black line) and stratified by self-identified ethnicity (Hispanic = orange, Non-Hispanic = purple). Correlation strengths did not differ significantly between ethnicity groups.

	Overall	Hispanic White	Non-Hispanic White	Sig.
**Concurrent Medications**				
Antihypertensive	57.6	59.5	54.9	.37
ACE Inhibitor	14.1	16.3	11.1	.15
Antiadrenergic Agent	8.8	7.0	11.1	.16
Beta-Blocker	23.9	26.5	20.4	.17
Calcium Channel Blocker	16.2	16.3	16.0	.95
Diuretic	14.1	14.4	13.6	.82
Vasodilator	1.3	1.4	1.2	.89
Angiotensin II Inhibitor	21.0	26.5	13.6	.002
Lipid Lowering	47.2	46.5	48.1	.75
Anticoagulant/Antiplatelet	34.7	33.0	37.0	.42
**Medical History**				
Heart Attack/Cardiac Arrest	6.5	8.0	4.4	.16
Atrial Fibrillation	3.5	2.8	4.4	.42
Angio/Endarterectomy/Stent	7.8	4.7	11.9	.01
Cardiac Bypass	4.3	4.7	3.8	.67
Pacemaker/Defibrillator	3.1	1.9	4.7	.13
Congestive Heart Failure	4.0	5.2	2.5	.20
Angina	9.6	7.7	12.2	.16
Stroke	3.0	3.3	2.5	.66
Transient Ischemic Attack	7.1	7.6	6.4	.67
Diabetes (T1 or T2)	19.1	22.6	14.4	.045
Hypertension	56.6	59.6	52.5	.17
Hypercholesterolemia	64.4	67.9	59.7	.10
Body Mass Index	27.4 (4.8)	28.3 (4.8)	26.4 (4.5)	<.001
Vascular Burden Score	2 (1-3), 1.7 (1.3)	2 (1-3), 1.8 (1.3)	2 (1-3), 1.6 (1.3)	.17

eTable 1: Descriptive characterization of reported concurrent medication use and medical history factors for the overall study cohort and stratified by ethnicity. All values are shown as percentages within the respective group except for body mass and the vascular burden score. Statistically significant group differences were assessed using chi-square (Mann-Whitney U for body mass index and vascular burden score). A modified vascular burden score was calculated based on prior publications (DeCarli et al., 2019)**)** as the sum of 7 possible vascular risk factors or diagnoses: cardiac-arrhythmias (atrial fibrillation OR defibrillator), coronary artery disease (angina OR angioplasty/endarterectomy/stent OR cardiac bypass OR heart attack), congestive heart failure, cerebrovascular disease (stroke OR transient ischemic attack), hypertension, hypercholesterolemia, diabetes (max score = 7; shown as median with interquartile range and mean with standard deviation in the table).

	Overall	Clinically Normal	Amnestic MCI	Amnestic Dementia	Non-Amnestic MCI/Dementia	Cognitively Impaired – Not MCI
**N**	379	62	179	58	48	32
**Age, years**	71.9 (7.8)	69.3 (5.9)	72.7 (7.9)	72.9 (10.5)	72.1 (5.7)	69.9 (6.0)
**Sex, %female**	228/379 (60.2)	50/62 (77.4)	92/179 (51.4)	37/58 (63.8)	26/48 (54.2)	25/32 (78.1)
**Education, years**	15.2 (3.5)	16.3 (2.8)	15.1 (3.5)	14.8 (3.7)	14.3 (4.0)	15.9 (3.2)
**Ethnicity, %Hispanic**	216/379 (57.0)	32/62 (51.6)	107/179 (59.8)	41/58 (70.7)	21/48 (43.8)	15/32 (46.9)
**CDR-Sum of Boxes**	2.1 (3.2)	0.1 (0.3)	1.4 (1.1)	7.5 (4.6)	1.9 (2.4)	0.7 (0.6)
**APOE e4, %carrier***	116/343 (33.8)	18/56 (32.1)	53/162 (32.7)	30/55 (54.5)	10/39 (25.6)	5/31 (16.1)
**MMSE**	26.7 (4.3)	29.3 (1.0)	27.4 (2.2)	20.0 (6.5)	26.9 (3.5)	29.1 (1.1)

*343/379 (91%) of the overall sample had *APOE* genotyping available

eTable 2: Descriptive characterization of the overall study cohort stratified by clinical diagnostic group. Final clinical diagnosis reflected multidisciplinary consensus based on neurological examination, neuropsychological evaluation, and other medical history data collected during the study visit. Data are presented as mean (standard deviation) unless otherwise noted.
